# Management of Arteriovenous Graft Infection

**DOI:** 10.3400/avd.oa.22-00058

**Published:** 2022-12-25

**Authors:** Yoichi Hisata, Taku Inoue, Yuichi Tasaki, Tomohiro Odate, Takafumi Yamada

**Affiliations:** 1 Division of Cardiovascular Surgery, Oita Prefectural Hospital, Oita, Oita, Japan; 2 Division of Cardiovascular Surgery, Sasebo Chuo Hospital, Sasebo, Nagasaki, Japan; 3 Division of Cardiovascular Surgery, Sasebo City General Hospital, Sasebo, Nagasaki, Japan; 4 Division of Cardiovascular Surgery, Nagasaki Harbor Medical Center, Nagasaki, Nagasaki, Japan

**Keywords:** arteriovenous graft infection, graft excision, reinfection

## Abstract

**Objective**: Arteriovenous graft (AVG) infection influences the survival and quality of life of patients, causing life-threatening sepsis reducing dialysis access. This study aimed to evaluate an appropriate treatment strategy for AVG infection.

**Methods**: We analyzed 61 cases involving AVG infections identified at a single center. The cases were divided into two groups based on the type of AVG and surgical methods, namely, currently used AVG (cAVG) (n=29) or abandoned AVG (aAVG) (n=32) and total graft excision (TGE) (n=10) or partial graft excision (PGE) (n=46).

**Results**: There was a significant difference in lower procedure frequency (p<0.001) and longer procedure time (p=0.014) in the cAVG group. A significant difference in lower reinfection rate (p=0.009) was found in the TGE group. Multivariable analysis confirmed that aAVG significantly independently affected the reinfection rate (hazard ratio, 2.208; 95% confidence interval, 1.069–4.561; p=0.032). *Staphylococcus aureus* was the most frequent cause of AVG infection (61.5%); 77.5% of *Staphylococcus aureus* were methicillin-resistant *Staphylococcus aureus*.

**Conclusion**: We found a higher risk of reinfection after PGE than TGE, and aAVG infection was associated with approximately two times higher likelihood of reinfection. These findings suggest that TGE should be considered for patients with AVG infections, particularly aAVG infections.

## Introduction

Compared with arteriovenous fistulas (AVFs), arteriovenous grafts (AVGs) are preferred as second-line treatment due to increased rates of infection and intervention, as well as inferior patency rates in patients with good superficial veins. AVG infection influences the survival and quality of life of patients with end-stage renal disease (ESRD) and leads to the loss of access to dialysis.^1)^

Managing AVG infection remains one of the most challenging and common infections faced by surgeons. The surgical management of AVG infection depends on the extent of the graft involvement and severity of sepsis. Purulent involvement of the entire graft with anastomotic disruption or systemic sepsis generally warrants total graft excision (TGE). For cases where only a graft segment is infected, partial graft excision (PGE), involving limited removal of only the infected portion of the graft with segmental reconstruction using uninfected tissue, can be performed.^2)^ The only definitive treatment for AVG infections is TGE. However, TGE is performed with complete graft removal, which creates a large open wound; the placement of temporary central venous access is recommended until the infection is controlled.^3)^ However, PGE with an interposition graft has been attempted to preserve vascular access and avoid the need for temporary access. PGE is considered a useful surgical method to achieve infection control and vascular access salvage in selected patients.^4)^ However, with careful selection of cases with well-incorporated graft remnants and aggressive resection of infected portions of the AVG, reinfection can be minimized. Selecting PGE is preferable for currently used AVG (cAVG) infections.

Data pertaining to the clinical presentation and treatment outcomes in patients with an AVG infection are limited. In addition, the use of TGE or PGE is controversial in AVG infection. AVG infection risk persists even when the graft is no longer in use (abandoned AVG [aAVG]). There has been some clinical research comparing TGE versus PGE, but relatively little between cAVG and aAVG.

This study aimed to evaluate the various types of treatment modalities for AVG infections. We compared the results of cAVG versus aAVG or TGE versus PGE to establish an appropriate treatment strategy for AVG infections.

## Methods

### Study design and participants

**Figure 1:**
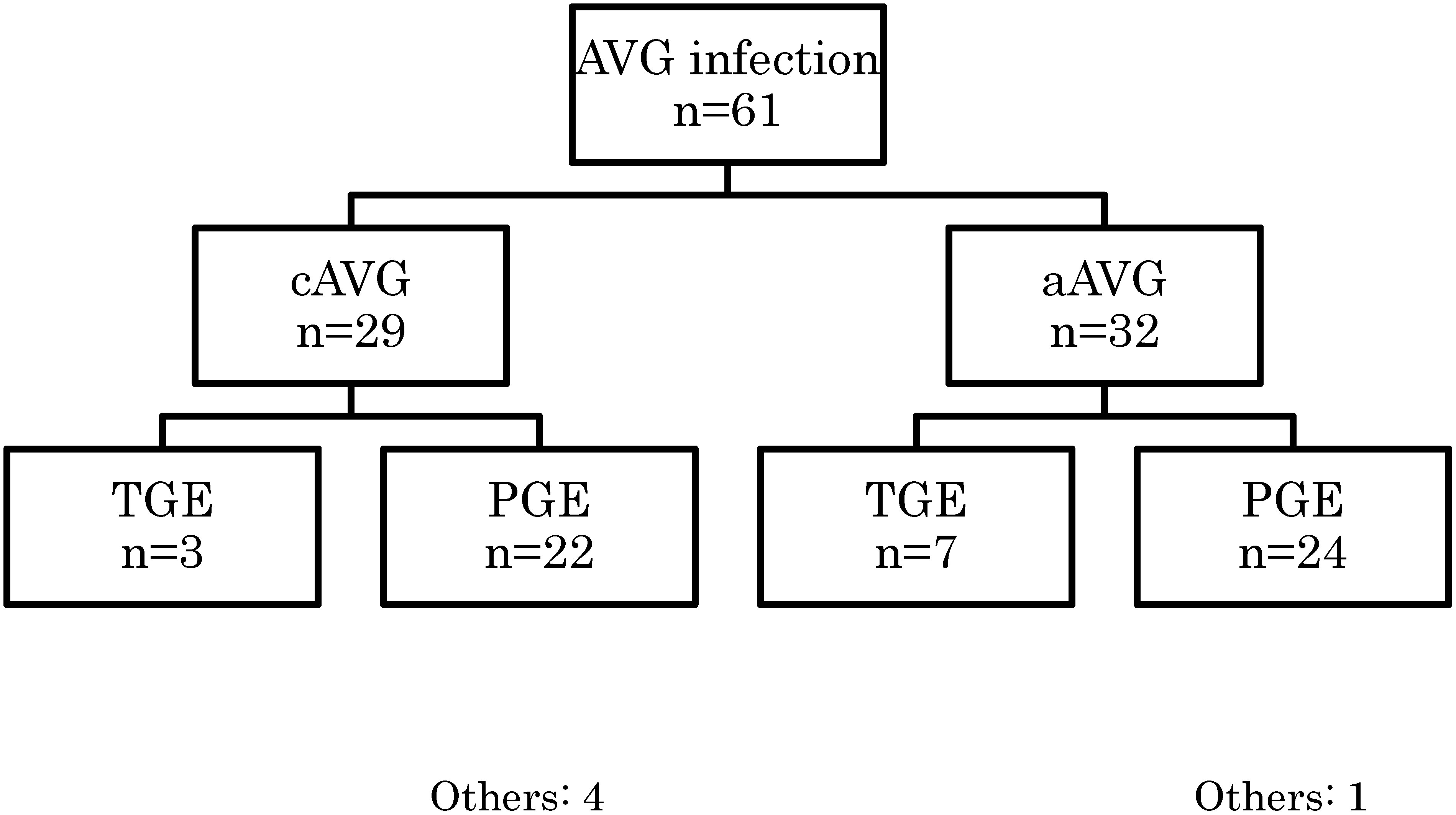
Fig. 1 Flowchart of patient selection.

AVG: arteriovenous graft; cAVG,:currently used arteriovenous graft; aAVG: abandoned arteriovenous graft; TGE: total graft excision; PGE: partial graft excision

This was a single-center, retrospective study of prospectively collected data on a cohort of 61 consecutive AVG infections in 32 patients requiring operation between January 1, 2013, and December 31, 2019, at our hospital. AVG infections were identified by clinical findings (clinical presentation was categorized as systemic sepsis, draining sinus tract, exposed graft, purulent drainage, erythema, pain overlying the graft, or hemorrhage), ultrasound examination, and cultures. The patients were divided into two groups: cAVG (n=29) and aAVG (n=32). The groups were subsequently subdivided into TGE (n=10) and PGE (n=46) (**Fig. 1**). Case notes were examined for operative details. Data were analyzed until cessation on August 30, 2020. The selections of TGE or PGE were dependent on the characteristics of the patient’s vasculature as assessed by each surgeon. The differences in baseline characteristics, procedural variables, and reinfection rate between the two groups were subsequently analyzed. The exclusion criterion was irrigation of the infection site. This study was approved by the institutional review board of our institution, and informed consent was not required for this study, as only de-identified data were used, and there was no contact with the patients. The primary end points were patients who were censored at the time of death, reinfection of AVGs, or surgical site infection.

### Statistical analyses

Descriptive statistics for all patient characteristics, risk factors, operative features, and postoperative outcomes were obtained using appropriate univariate hypothesis testing. Student’s t-tests and Chi-square tests were used to compare the averages of continuous and categorical variables, respectively, between the groups. Continuous data are represented as either mean±standard deviation for normally distributed variables or median for non-normally distributed variables. Reinfection rate was obtained using the Kaplan–Meier method. The differences between the groups were analyzed using log-rank tests. Cox proportional hazards modeling of categorical variables were performed to analyze the concurrent effects of various factors on reinfection rates. Hazard ratio (HR) and 95% confidence intervals (CIs) were calculated. A p value of 0.05 indicated statistical significance. Analyses were performed using IBM SPSS statistics version 25 (IBM Corp., Armonk, NY, USA).

## Results

We identified 61 infected AVGs in 32 patients. There were 29 (47.5%) and 32 (52.5%) AVGs in the cAVG and aAVG groups, respectively. The mean follow-up period was 33.0±23.3 months. **Table 1** summarizes the patient characteristics and perioperative data. The patient’s mean age and sex distribution were similar in the two groups. The cAVG group had a higher body mass index and a higher rate of dyslipidemia. Regarding the cause of renal failure, the rate of nephrosclerosis was significantly higher in the cAVG group than in the aAVG group. Diabetes mellitus (DM) was the most common cause of renal failure requiring hemodialysis in both groups, and the incidence of DM did not significantly differ between the groups. Procedure (TGE or PGE) did not significantly differ between the groups. However, significant differences were noted in higher rate of initial operation (86.2% vs. 40.6%; p<0.001), lower frequency of operation (1.13±0.35 times vs. 2.00±1.02 times; p<0.001), and longer procedure time (102.9±57.0 min vs. 70.1±43.7 min; p=0.014) in the cAVG groups. However, the incidence of reinfection did not significantly differ between the groups (31.0% vs. 40.6%; p=0.436) (**Table 1A**).

**Table 1A:** Table 1A Baseline patient characteristics of the cAVG and aAVG groups

Variable	cAVG (n=29)	aAVG (n=32)	p value
Patients			
Age, years	74.2±9.0	70.5±10.8	0.152
Female, no. (%)	17 (58.6)	20 (62.5)	0.756
Weight, kg	47.6±10.9	43.5±10.3	0.141
Height, m	1.49±0.13	1.51±0.08	0.360
BMI, kg/m^2^	25.9±3.9	23.0±3.9	0.005
Coexisting disease			
Hypertension, no. (%)	28 (96.6)	28 (87.5)	0.198
DM, no. (%)	13 (44.8)	16 (50.0)	0.686
DM on insulin, no. (%)	7 (24.1)	3 (9.4)	0.120
Dyslipidemia, no. (%)	6 (20.7)	0 (0.0)	0.006
Cause of renal failure			
DM, no. (%)	10 (34.5)	11 (34.4)	0.993
Nephrosclerosis, no. (%)	7 (24.1)	0 (0.0)	0.003
Chronic glomerulonephritis, no. (%)	2 (6.9)	4 (12.5)	0.463
Unknown, no. (%)	7 (24.1)	9 (28.1)	0.724
Others, no. (%)	3 (10.3)	8 (25.0)	0.137
Laboratory data			
White-cell count (10^3^/µL)	6.19±2.53	5.65±2.23	0.406
CRP (mg/dL)	3.22±3.29	3.98±6.00	0.569
Number of operations			
First time, no. (%)	25 (86.2)	13 (40.6)	p<0.001
Number of operations, n	1.13±0.35	2.00±1.02	p<0.001
Procedure			
Procedure time, min	102.9±57.0	70.1±43.7	0.014
TGE, no. (%)	3 (10.3)	7 (21.9)	0.300
Outcome			
Reinfection, no. (%)	9 (31.0)	13 (40.6)	0.436

BMI: body mass index; DM: diabetes mellitus; CRP: C-reactive protein; cAVG: currently used arteriovenous graft; aAVG: abandoned arteriovenous graft; TGE: total graft excision

**Figure 2:**
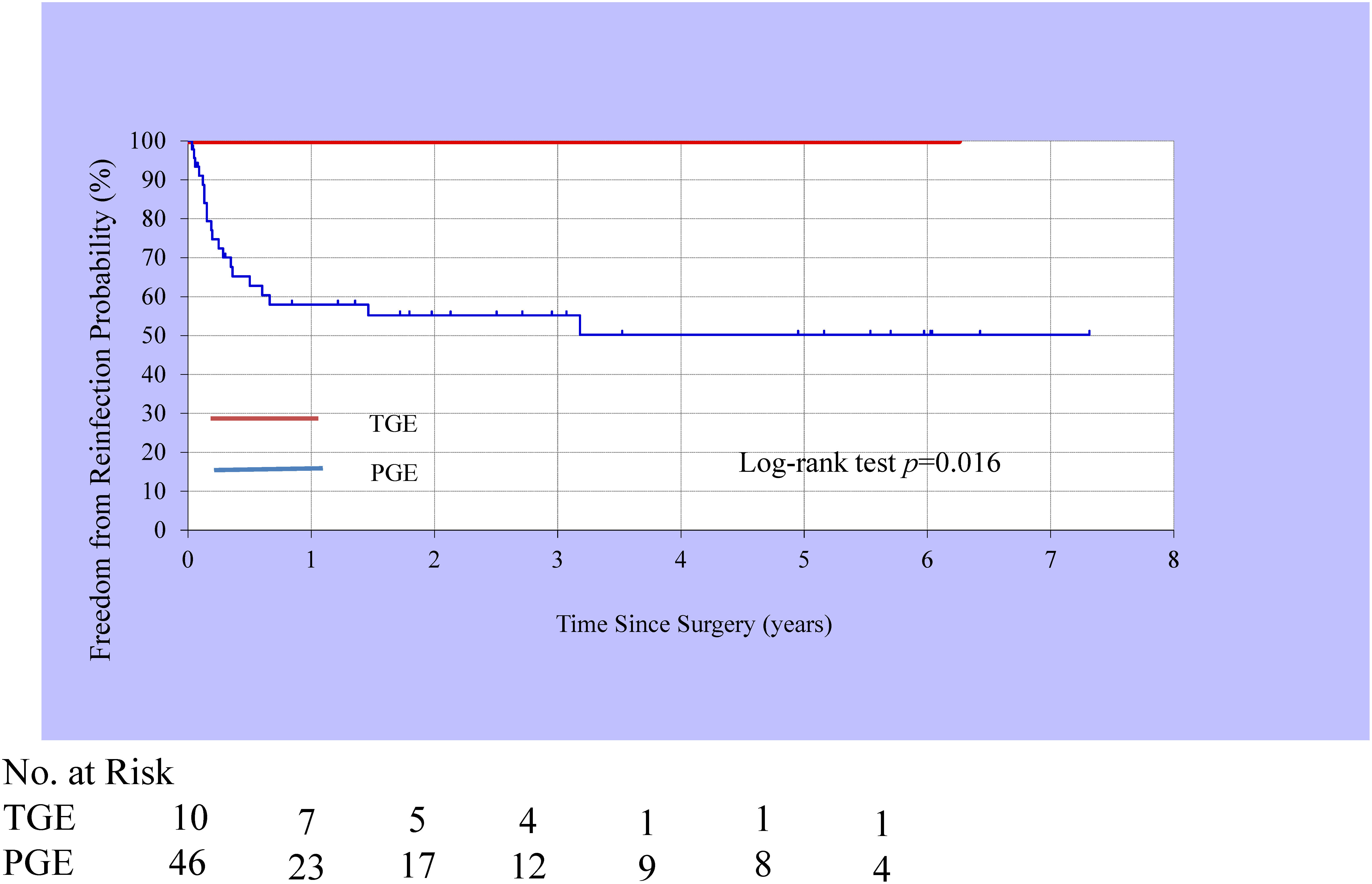
Fig. 2 Kaplan–Meier analysis of the freedom from reinfection probability between the groups.

TGE: total graft excision; PGE: partial graft excision

**Table 1B** shows comparison of the characteristics and the perioperative data between TGE and PGE. The mean age of the patients and sex distribution were similar in the two groups. The rate of initial operation, frequency of operation, and procedure time did not significantly differ between the groups. However, the incidence of reinfection was significantly higher in the PGE group (0.0% vs. 43.5%; p=0.009). The freedom from reinfection probability rates at 1 and 5 years were both 100% for the TGE group compared with 58.0% and 50.2% for the PGE group, respectively, which is significantly higher in the TGE group (p=0.016) (**Fig. 2**).

**Table 1B:** Table 1B Baseline patient characteristics of the TGE and PGE groups

Variable	TGE (n=10)	PGE (n=46)	p value
Patients			
Age, years	71.1±9.8	73.0±10.3	0.599
Female, no. (%)	5 (50.0)	17 (37.0)	0.444
Weight, kg	46.6±9.1	44.7±11.2	0.614
Height, m	1.50±0.06	1.50±0.12	0.822
BMI, kg/m^2^	24.9±3.6	24.0±4.4	0.548
Coexisting disease			
Hypertension, no. (%)	10 (100.0)	41 (89.1)	0.275
DM, no. (%)	7 (70.0)	21 (45.7)	0.163
DM on insulin, no. (%)	3 (30.0)	7 (15.2)	0.269
Dyslipidemia, no. (%)	1 (90.0)	3 (6.5)	0.699
Cause of renal failure			
DM, no. (%)	6 (60.0)	14 (30.4)	0.077
Nephrosclerosis, no. (%)	0 (0.0)	6 (13.0)	0.227
Chronic glomerulonephritis, no. (%)	1 (10.0)	5 (10.9)	0.936
Unknown, no. (%)	0 (0.0)	15 (32.6)	0.035
Others, no. (%)	3 (30.0)	6 (13.0)	0.186
Laboratory data			
White-cell count (10^3^/µL)	6.54±1.35	5.85±2.61	0.448
CRP (mg/dL)	4.88±7.50	3.48±4.23	0.440
Number of operations			
First time, no. (%)	6 (60.0)	28 (60.9)	0.959
Numbers of operation, n	1.80±1.14	1.57±0.89	0.473
Procedure			
Procedure time, min	78.4±48.5	94.0±52.1	0.390
Outcome			
Reinfection, no. (%)	0 (0.0)	20 (43.5)	0.009

BMI: body mass index; DM: diabetes mellitus; CRP: C-reactive protein; TGE: total graft excision; PGE: partial graft excision

The most common infectious agents in AVGs were *Stapylococcus aureus* (61.5%) and *Serratia marcescens* (4.6%) in gram-positive and gram-negative bacteria, respectively. The most frequent species was methicillin-resistant *Staphylococcus aureus* (MRSA, 47.7%); 77.5% of *S. aureus* were MRSA (**Table 2**).

**Table 2:** Table 2 Microbiology of AVG infection

Microbiology	n=65
Gram-positive bacteria	
MRSA	31
MSSA	9
*Staphylococcus epidermidis*	6
*Staphylococcus lugdunensis*	2
*Enterococcus faecium*	2
Aerobic gram-positive bacilli	2
α-*Streptococcus*	1
*Peptostreptococcus prevotii*	1
*Corynebacterium jeikeium*	1
*Staphylococcus caprae*	1
*Enterococcus faecalis*	1
Anaerobic gram-positive bacilli	1
Gram-negative bacteria	
*Serratia marcescens*	3
*Enterobacter aerogenes*	2
*Campylobacter species*	1
*Proteus mirabilis*	1

AVG: arteriovenous graft; MRSA: methicillin-resistant *Staphylococcus aureus*; MSSA: methicillin-sensitive *Staphylococcus aureus*

Multivariate analysis using Cox proportional hazards models revealed that aAVG was the independent variable that had a statistically significant effect on reinfection rates (HR, 2.208; 95% CI, 1.069–4.561; p=0.032). aAVG was associated with an approximately twofold higher likelihood of reinfection. However, a statistical difference was not noted between the TGE group and MRSA (**Table 3**).

**Table 3:** Table 3 The effect of independent variables on the reinfection rate

Variable	Hazard ratio	95% confidence interval	p value
Age >65 years	1.419	0.602–3.344	0.424
Male	1.694	0.769–3.731	0.191
BMI >23 kg/m^2^	0.569	0.248–1.305	0.183
DM	1.488	0.711–3.114	0.291
Procedure time >1 h	1.051	0.473–2.339	0.902
aAVG	2.208	1.069–4.561	0.032
TGE	1.304	0.614–2.772	0.490
MRSA	1.219	0.625–2.379	0.562

BMI: body mass index; DM: diabetes mellitus; aAVG: abandoned arteriovenous graft; TGE: total graft excision; MRSA: methicillin-resistant *Staphylococcus aureus*

## Discussion

The “Fistula First” initiative recommends AVFs as the preferred vascular access method for dialysis due to the associated high functional patency rates and low complication rates.^5–9)^ Particularly compared to AVF, AVG usage was associated with a fivefold increase in infection.^10)^ AVG infections influence the survival and quality of life of patients with ESRD and loss of dialysis access.^1)^ Previous studies have evaluated whether TGE and PGE are appropriate treatment methods for AVG infection.^4,11–13)^ However, the characteristics and effect on reinfection rates between cAVG and aAVG or TGE and PGE have not been compared. This study compared the results of cAVG and aAVG or TGE and PGE, to establish an appropriate treatment strategy for AVG infections. Our results showed that TGE was associated with lower reinfection probability rates and multivariate analysis showed that aAVG was associated with an approximately two times higher likelihood of reinfection. However, there was no statistical difference between the TGE group and MRSA.

The risk of AVG infection in hemodialysis (HD) patients does not end when the graft is no longer in use, such as aAVG. It is common practice to leave these aAVG in place, and thus, many HD patients have one or more old aAVG in their extremities. Although these aAVG were innocuous, it was recently recognized that they may harbor occult bacterial infection that can lead to serious infectious complications; thus, aAVG has been termed “A Silent Source of Infection.”^14,15)^ Although there is a clear indication for removal of cAVG infections in unresolved bacteremia, there are no available data in the literature regarding the incidence of aAVG infections and their management.^15)^ Schild et al. reported that 23% of AVG infections were aAVG.^16)^ Our data showed the aAVG infection rate to be 52.5%; compared with cAVG, significant differences were noted for lower rate of initial operation, increased frequency of operation, and shorter procedure time in the aAVG group. Longer procedure time in the cAVG group may be because the clump of artery or AVG was required for the surgery. Reinfection rate did not significantly differ; however, the multivariate analysis revealed that aAVG was associated with an approximately two times higher likelihood of reinfection. It suggested that TGE was the more appropriate procedure for the aAVG group.

Purulent involvement of the entire graft with anastomotic disruption or systemic sepsis generally warrants TGE. For stable individuals with total graft involvement but an intact, uninfected, well-incorporated anastomosis, subtotal graft excision (SGE), which retains an oversewn stump of the prosthesis on the originating artery, is typically appropriate. For cases in which only a segment of the graft is infected, PGE can be performed.^2)^ Ryan et al. recommended TGE when patients present with sepsis or the entire graft was bathed in pus, SGE when the entire graft was removed except a small oversewn cuff of prosthetic material on an underlying artery, and PGE when only a limited infected portion of the graft was removed and a new graft was rerouted in adjacent sterile tissue to maintain patency of the original graft. They reported that their strategy has proven highly successful in the management of these complicated cases.^3)^ In our hospital, the selection of TGE (n=10, 17.9%) or PGE (n=46, 82.1%) was dependent on the characteristics of the patient’s vasculature, as assessed by each surgeon, and SGE was not performed.

The only definitive treatment for AVG infection is TGE. In several previous studies, TGE was found to have virtually no incidence of reinfection when compared to other methods.^11,16)^ Our results also showed that there were no cases of reinfection in the TGE group. However, the disadvantage of TGE is the possibility of arterial occlusion, increased risk of median nerve injury, and arterial hemorrhage. Furthermore, TGE also mandates the placement of a temporary dialysis catheter for a few months, which leads to prolonged and repeated hospitalization and healthcare costs. In addition, the catheter itself carries a higher risk of recurrent infection.^4)^ Our multivariate analysis showed that there were no statistical differences in the factors in the TGE group on reinfection rates.

However, Bhat et al. first introduced graft preservation techniques, which remain controversial.^17)^ The main advantages of PGE are the maintenance of the patency of the originating artery, avoidance of a potentially morbid dissection, and that incorporated portions of the graft are available for immediate dialysis without a temporary dialysis catheter. In addition, they reported that if the infection was localized, PGE with rerouting the new graft in adjacent sterile tissue could be safely performed with an expected success rate approaching 80%.^18)^ However, a major drawback of PGE is the potential for reinfection in the remnant portion of graft.^3)^ Several studies have shown an increased probability of reinfection after PGE compared with TGE for AVG infection.^13,19)^ Previous studies reported that the reinfection rates were 46.7% for PGE with bypass, 14.3% for PGE without bypass and no TGE at the excision site, and 8% for PGE.^20,21)^ Schutte et al. also reported that the local reinfection rate was higher in the PGE group (19.8% vs. 0%).^11)^ Our study revealed that reinfection occurred in 43.5% of the PGE group and 0% of the TGE group. Moreover, the freedom from reinfection rate was significantly better in the TGE group. With careful selection of cases with well-incorporated graft remnants and aggressive resection of infected portions of the AVG, infection recurrence can be minimized.^3)^ Our reinfection rate was significantly higher than previous reports. This result may be caused by inadequate patient selection and insufficient rejection of AVG infection. Some surgeons may have selected PGE purely to avoid the troublesome TGE procedure, leading to reinfection and repeated PGE procedures. Our data suggested that we should have selected TGE for AVG infection, particularly for aAVG infection.

Since normal skin flora is predominantly composed of gram-positive organisms, AVG infection is generally caused by gram-positive bacteria, particularly strains of *Staphylococcus aureus*, which are documented in as many as 2.0–68.6% of infections. Gram-negative organisms are less frequent and can be found in less than 1–43.1% of infections.^2,3,22)^ Our results also indicated that *Staphylococcus aureus* (61.5%) was the most common infectious agent in AVGs and gram-negative bacteria only made up 10.8% of infections. MRSA is a growing problem for dialysis centers, and MRSA in patients with catheter-related bacteremia reported incidences from 7.1 to 62.8%.^22)^ The most frequent species in this study was MRSA (47.7%). Our multivariate analysis showed that MRSA was not associated with reinfection.

This study has several limitations. First, this was a single-center, retrospective, and non-randomized study. Thus, the preoperative characteristics differed between the two groups. Second, other outcome risk factors, such as repeat cannulation, poor personal hygiene, increased number of hospitalizations, increased duration of graft use, increased age, DM, access site, and ambulatory limitation, were not analyzed. Third, the number of patients was small and sampling bias may have been introduced. Fourth, the selection of TGE or PGE was dependent on the characteristics of the patient’s vasculature as assessed by each surgeon and was subject to biases from unobserved differences. Finally, the mean follow-up duration was 33.0±23.3 months. Thus, long-term studies focused on the patency rates and vascular access complications are warranted.

## Conclusion

Due to a higher risk of reinfection after PGE, multivariable analysis revealed that aAVG infections were associated with an approximately two times higher likelihood of reinfection. Therefore, our data suggest that TGE should be considered for patients with AVG infections, particularly aAVG infections.
